# Ethanol-Induced Face-Brain Dysmorphology Patterns Are Correlative and Exposure-Stage Dependent

**DOI:** 10.1371/journal.pone.0043067

**Published:** 2012-08-22

**Authors:** Robert J. Lipinski, Peter Hammond, Shonagh K. O’Leary-Moore, Jacob J. Ament, Stephen J. Pecevich, Yi Jiang, Francois Budin, Scott E. Parnell, Michael Suttie, Elizabeth A. Godin, Joshua L. Everson, Deborah B. Dehart, Ipek Oguz, Hunter T. Holloway, Martin A. Styner, G. Allan Johnson, Kathleen K. Sulik

**Affiliations:** 1 The Bowles Center for Alcohol Studies, University of North Carolina, Chapel Hill, North Carolina, United States of America; 2 Institute of Child Health, University College London, London, United Kingdom; 3 Department of Psychiatry, University of North Carolina, Chapel Hill, North Carolina, United States of America; 4 Center for In Vivo Microscopy, Duke University, Durham, North Carolina, United States of America; 5 Department of Cell and Developmental Biology, University of North Carolina, Chapel Hill, North Carolina, United States of America; Ecole Normale Supérieure de Lyon, France

## Abstract

Prenatal ethanol exposure is the leading preventable cause of congenital mental disability. Whereas a diagnosis of fetal alcohol syndrome (FAS) requires identification of a specific pattern of craniofacial dysmorphology, most individuals with behavioral and neurological sequelae of heavy prenatal ethanol exposure do not exhibit these defining facial characteristics. Here, a novel integration of MRI and dense surface modeling-based shape analysis was applied to characterize concurrent face-brain phenotypes in C57Bl/6J fetuses exposed to ethanol on gestational day (GD)7 or GD8.5. The facial phenotype resulting from ethanol exposure depended upon stage of insult and was predictive of unique patterns of corresponding brain abnormalities. Ethanol exposure on GD7 produced a constellation of dysmorphic facial features characteristic of human FAS, including severe midfacial hypoplasia, shortening of the palpebral fissures, an elongated upper lip, and deficient philtrum. In contrast, ethanol exposure on GD8.5 caused mild midfacial hypoplasia and palpebral fissure shortening, a shortened upper lip, and a preserved philtrum. These distinct, stage-specific facial phenotypes were associated with unique volumetric and shape abnormalities of the septal region, pituitary, and olfactory bulbs. By demonstrating that early prenatal ethanol exposure can cause more than one temporally-specific pattern of defects, these findings illustrate the need for an expansion of current diagnostic criteria to better capture the full range of facial and brain dysmorphology in fetal alcohol spectrum disorders.

## Introduction

Prenatal ethanol exposure causes a range of structural and functional abnormalities, collectively termed fetal alcohol spectrum disorder (FASD). At the severe end of this spectrum is fetal alcohol syndrome (FAS), which is characterized by growth retardation, CNS dysfunction, and a specific pattern of craniofacial dysmorphology. While prenatal ethanol exposure is already recognized as a leading known cause of mental disability [1], the number of affected individuals is undoubtedly underestimated, due in large part, to difficulty in making an appropriate diagnosis [2].

Development of the face and brain is intimately interrelated, as the brain provides structural, cellular, and molecular input that the guides development of the adjacently developing face [3], [4], [5], [6]. This relationship is exploited in clinical diagnostics, as syndromes involving primary brain abnormalities can often be recognized by associated facial dysmorphism [7]. Whereas CNS abnormalities are the most disabling manifestation of FAS, current diagnostic approaches are directed at the recognition of a specific pattern of craniofacial dysmorphology, including reduced head circumference, shortened palpebral fissures, and an upper lip with a thin vermillion border and relatively indistinct philtrum [8], [9]. However, as determined through confirmed maternal ethanol consumption and measureable neurobehavioral deficits, a large proportion of children affected by prenatal ethanol exposure do not or only partially express diagnostic facial features [8], [10]. Failure to identify ethanol-affected individuals represents a major hindrance to the effective implementation of appropriate and timely interventions for FASD [11].

**Figure 1:**
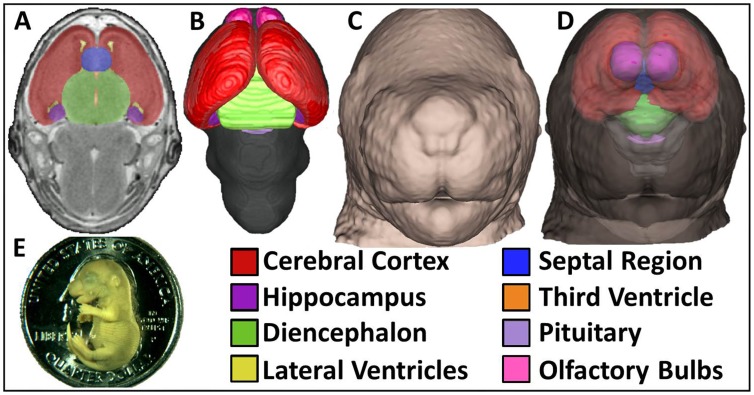
MRM enables concurrent 3D analyses of the brain and face of GD17 mouse fetuses. Forebrain and pituitary regions were manually segmented from transverse 39 µm MRM sections (A). 3D brain reconstructions were generated by overlaying manually segmented regions with whole-brain masks (B). From the same MRM scans, 3D head reconstructions were created featuring detailed facial surfaces (C). The brain and face can be visualized concurrently *in situ* by reducing head surface opacity (D). The size of a GD17 mouse fetus can be appreciated when shown in scale with a U.S. quarter dollar coin (E).

Animal-based studies have been instrumental in elucidating the complex pathogenesis of ethanol-mediated teratogenicity [12], [13], [14], [15], allowing for precise control of critical variables, such as genetic background, maternal nutritional status, and timing and dose of administration. Highlighting the importance of timing of exposure, Dunty et al. identified stage-specific cell populations selectively vulnerable to ethanol-induced apoptosis in the developing brain and face of the mouse [16]. Accordingly, Sulik et al. found that the critical window of exposure for induction of the classic facial features of FAS is limited to early gastrulation stages of development (gestational day [GD] 7 in the mouse) [17]. More recently, unique patterns of regional brain abnormalities were found to result from stage-specific ethanol exposure occurring between GD7 and GD10 [18], [19], [20]. These results clearly demonstrate a temporal dependence in the manifestation of ethanol-induced abnormalities. However, no study to date has provided objective and quantitative comparisons of the concurrent face and brain abnormalities which result from stage-specific ethanol insult.

**Figure 2:**
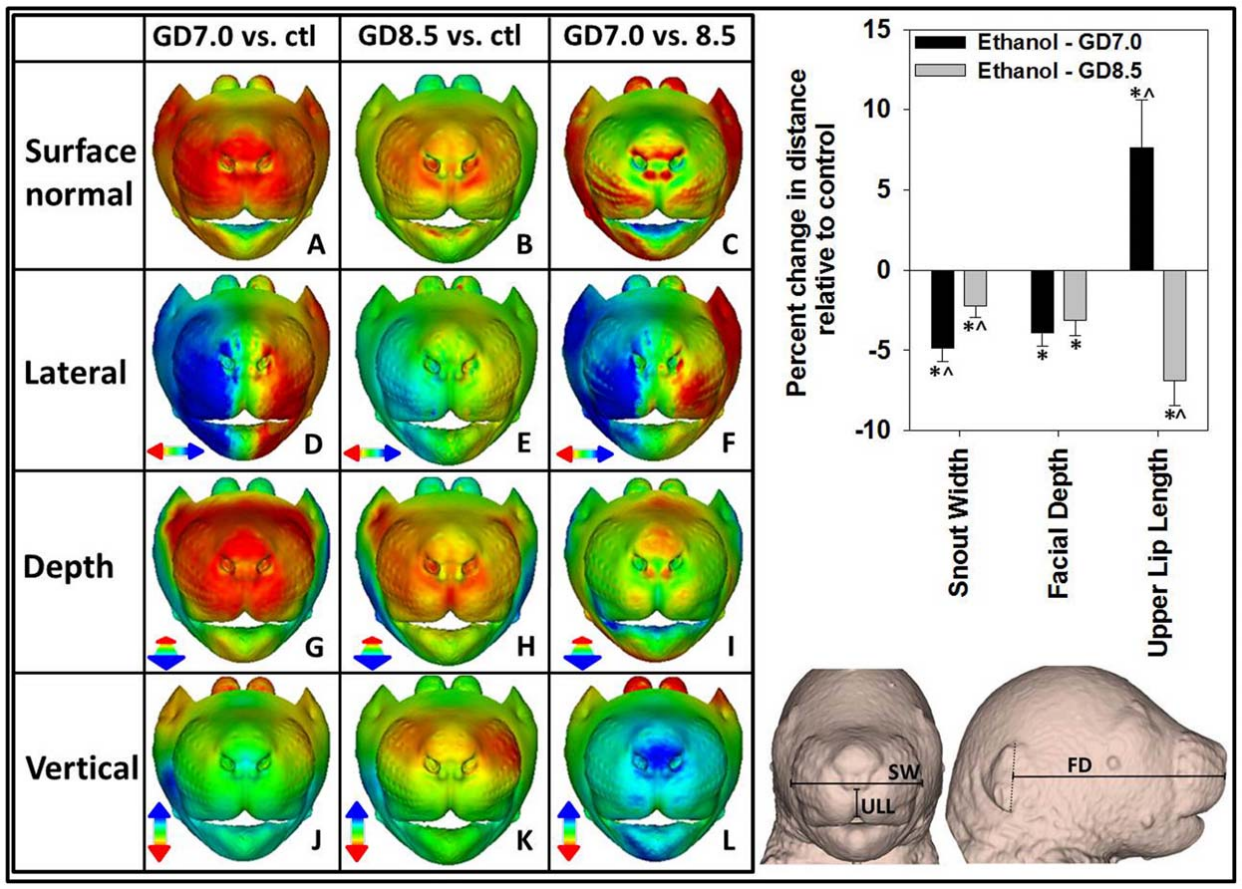
DSM illustrates unique facial phenotypes resulting from stage-specific ethanol exposure. (Left) Mean surface shape of the GD7 and GD8.5 exposure groups relative to the control group is shown in the first and second columns respectively, while the GD7 group is directly compared to the GD8.5 ethanol-exposed group in the third column. (A–C) Color-map comparisons reflecting the displacement of mean surface shape for the indicated groups, where red indicates regions most distant and internal, while blue indicates regions most distant and external. Other colors shown in the scales identify intermediate positions. (D–L) Color-map comparisons reflecting the displacement of the indicated mean surface shapes parallel to the three orthogonal axes. Red and blue color intensities reflect displacement in the direction indicated by the corresponding color-coded arrow. Changes are shown at a scale of 1.2 standard deviations. (Right) Snout width (SW) was measured between the most lateral 3^rd^ row of vibrissae; Median upper lip length (ULL) was measured from the lower edge of the nostrils to the bottom of the upper lip; Facial depth (FD) was measured from the middle of the ear to the top of the philtrum. Values represent the means + the S.E.M. ^*^p<0.05 compared to control group.^ ∧^p<0.05 compared to counterpart ethanol exposure group.

The studies described herein applied a novel integration of high-resolution MRI - magnetic resonance microscopy (MRM) - and dense surface modeling (DSM)-based shape analysis to define and compare the face/brain sequelae resulting from acute ethanol insult occurring during early gastrulation (GD7) or neurulation (GD8.5) stages of embryogenesis in mice. These stages of exposure were chosen because they have been shown to cause unique patterns of cell death in the developing forebrain [16], [21]. While applied independently, clinical research efforts have also used MRI to define brain dysmorphology [22], [23], [24], [25], and DSM has been utilized to describe facial phenotypes in syndromes involving co-occurring brain abnormalities [7], [26], [27], [28], [29]. Concerted application of these technologies to a well-established model of FASD facilitated an experimental capacity not possible in human populations, while providing findings that can be readily extrapolated to the clinical setting.

**Figure 3:**
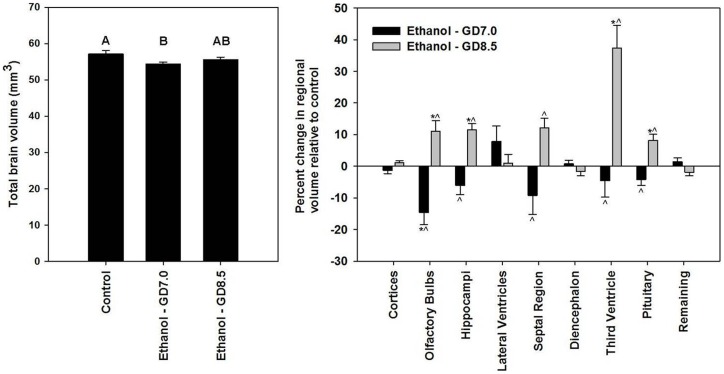
Volumetric brain measurements demonstrate unique brain abnormalities resulting from stage-specific ethanol exposure. (Right) Total brain volumes were derived from automated skull stripping. Values represent the mean + S.E.M. Letters above each bar indicate group classes; the same letter above a subset of bars denotes lack of statistical difference, whereas different letters represent statistically different classes (p<0.05). (Right) For determination of disproportionate differences, the volume of each manually segmented forebrain region was calculated as a percentage of total brain volume for each animal. Remaining volume includes mid- and hindbrain regions. To illustrate relative changes on the same scale, percent volumes are normalized to mean control values. Values represent the mean ± the S.E.M. ^*^p<0.05 compared to control group. ^∧^p<0.05 compared to counterpart ethanol exposure group.

## Results

For examination of dysmorphology patterns resulting from stage-specific insult, GD17 C57Bl/6J fetuses exposed to ethanol on GD7 or GD8.5, and vehicle-exposed controls, were imaged by MRM (Fig 1). This allowed extraction of 3D facial surfaces as well as segmentation of forebrain regions in a randomly selected subpopulation of fetuses. Focus was placed on the forebrain because its development is particularly reciprocative with that of the face [5]. By establishing a correspondence of over 65,000 points across each image, DSM was used to generate objective comparisons and dramatic visualization of mean face and brain shape differences in each ethanol exposure group relative to control fetuses. To highlight the distinct nature of resulting phenotypes, direct comparison between the ethanol exposure groups was also made. Between-group size differences were highlighted by comparison of displacement normal to the surface where red/green/blue coloring indicate where the normalized surface shape is contracted/coincident/expanded. To further dissect morphological differences, comparisons of surface displacement parallel to the three orthogonal axes were also generated, where coloring illustrates corresponding shifts in the direction indicated by accompanying arrows.

### Ethanol-induced Facial Dysmorphology

The comparisons shown in Fig. 2 highlight unique patterns of facial dysmorphology in the two ethanol exposure groups. For the GD7 ethanol exposure to control comparison, dark red coloring surrounding the eyes represents a marked contraction of the sides of the head indicative of microcephaly (Fig. 2A), while similar coloring of the snout indicates severe midfacial hypoplasia. In the GD8.5 ethanol exposure to control comparison, a unique pattern of red/yellow coloring of the snout is seen (Fig. 2B), suggesting a distinct form of midfacial hypoplasia which is distinguishable when the GD7 and GD8.5 ethanol exposure groups are directly compared (Fig. 2C).

Along the lateral axis, severe midfacial narrowing is apparent in the GD7 group (Fig. 2D), while a more modest narrowing, particularly in the lower snout is evident in the GD8.5 exposure group (Fig. 2E, F). Reduced midfacial depth is visible in both groups compared to controls, such that most of the snout surface appears coincident when they are directly compared (Fig. 2G–I). In the vertical dimensions, a left-side dominant ventral displacement of the upper snout is unique to the GD8.5 exposure group (Fig. 2K). Mandibular hypoplasia (micrognathia) that is more severe in the GD7 exposure group is evident in both the lateral and vertical dimension (Fig. 2D, J). Dynamic morphs between the mean control and ethanol-exposed faces in portrait and profile views provide dramatic illustration of differences in facial shape between the two ethanol exposure groups (Fig. S1). Significant differences in snout width and midfacial depth relative to controls were verified using simple linear measurements (Fig. 2, right).

**Figure 4:**
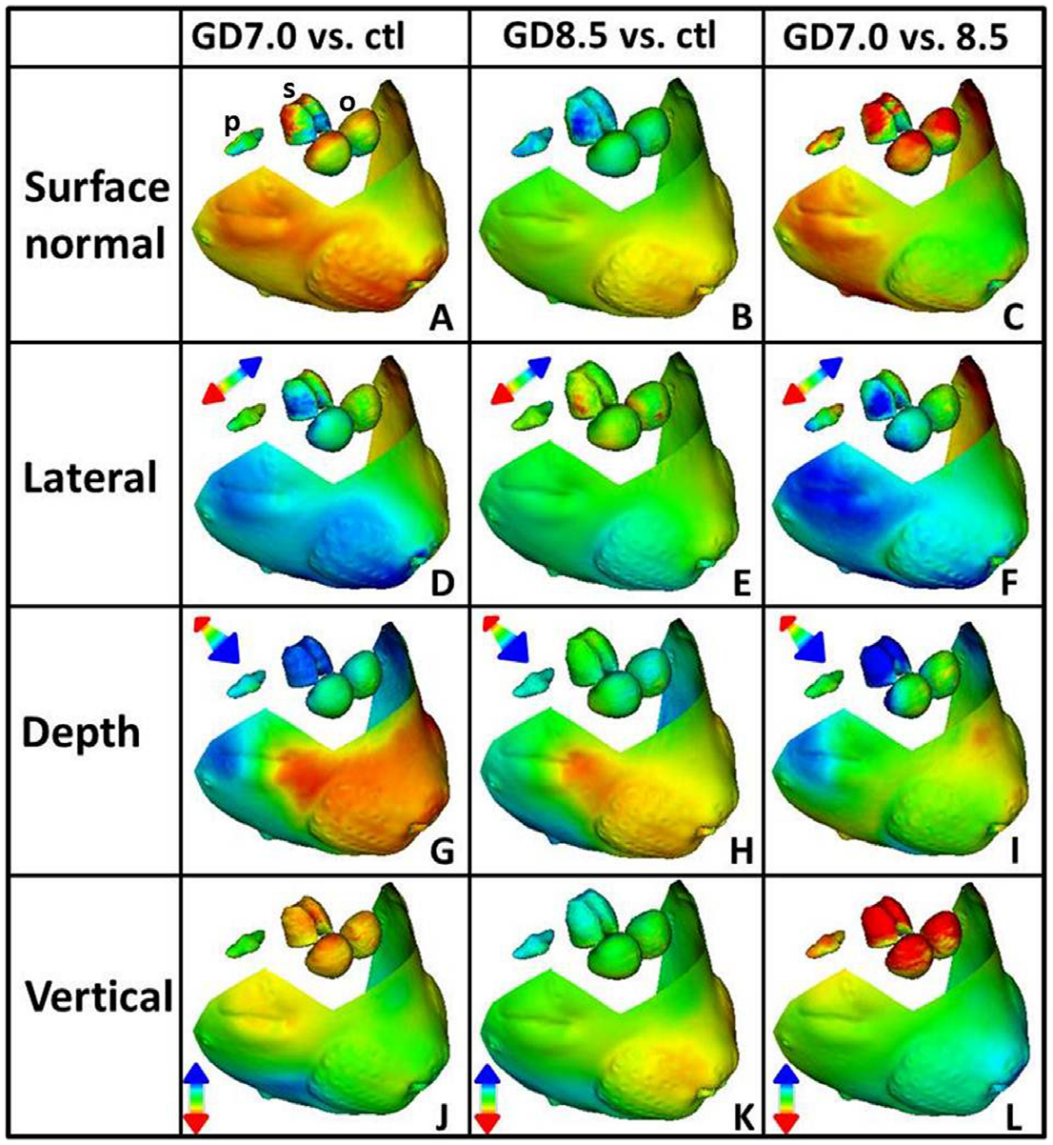
DSM illustrates unique brain and face phenotypes resulting from stage-specific ethanol exposure. Mean shape comparisons are shown for the facial surface along with the olfactory bulbs (o), septal region (s), and pituitary (p). Comparisons parallel those shown in Fig. 2. Changes are shown at a scale of 1.5 standard deviations. In this anterior-oblique view, differences in palpebral fissure length are readily apparent.

Group differences in the shape of the upper lip midline and nose were also apparent. In the GD7 ethanol exposure group, depth reduction of the upper lip midline groove is less than that of the surrounding areas of the upper lip, indicative of a deficient philtrum (Fig. 2G). In the GD8.5 exposure group, the philtrum appears distinct and the upper lip midline is more reduced in depth than the surrounding area (Fig. 2H). Vertical changes in the placement of the lower aspect of the nose are also apparent, where a superior displacement of the columella can be seen following GD7 exposure (Fig. 2J), while inferior displacement is seen in the GD8.5 exposure group (Fig. 2K). These opposing changes are highlighted by robust superior displacement of the lower aspect of the nose when the GD7 group is compared directly with the GD8.5 group (Fig. 2L). While the exposure groups exhibit opposing displacement of the lower aspect of the nose, green coloring of the lower aspect of the upper lip midline indicates coincident positioning, thus suggesting that unique differences in upper lip length exist between the exposure groups. Linear measurements confirmed that median upper lip length was significantly increased in the GD7 exposure group, and significantly reduced in the GD8.5 exposure group, relative to controls (Fig. 2, right).

**Figure 5:**
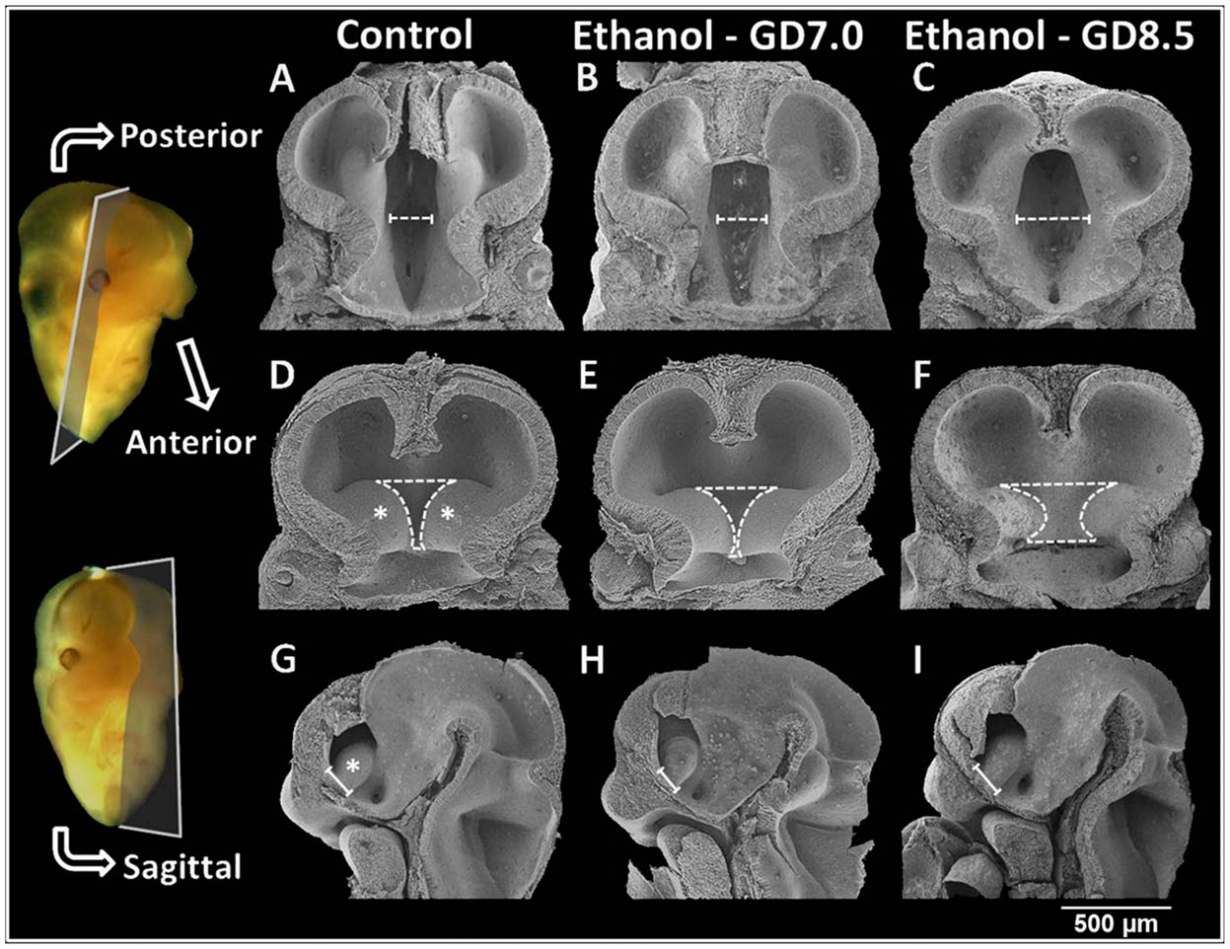
Scanning electron microscopy illustrates unique brain malformations in GD12 embryos exposed to ethanol at GD7 or GD8.5. (A–F) Images of specimens hemisected in the coronal (frontal) plane illustrate posterior and anterior aspects of the embryonic brain in control and ethanol-affected groups. (G–I) Additional GD12 embryos were cut to provide a sagittal view of the brain. Notable abnormalities include differences in width of the third ventricle (dashed calipers), and the area from which the septal region will develop (dashed outline in the anterior view and solid calipers in sagittal view). Ganglionic eminences (*).

Comparisons of mean facial surfaces also highlighted differences in palpebral fissure length between treatment groups. In the GD7 ethanol exposure group, red coloring of the area anterior-medial to both eyes (endocanthus) is indicative of markedly reduced depth compared to controls (Fig. 2G). The GD8.5 ethanol exposure group shows a similar but more subtle depth reduction (Fig. 2H). In both groups, depth reductions surrounding the eye appear right-side dominant. In both clinical populations and animal model systems reduced palpebral fissure length is associated with ocular abnormalities [30], [31], [32]. Using a previously-described classification scheme [30], ocular phenotypes were examined in the entire population of ethanol-exposed and control groups. Compared to controls, the incidence and severity of ocular defects was markedly increased in animals exposed to ethanol at GD7, while a more modest increase was observed in the population exposed at GD8.5 (Fig. S2).

### Ethanol Induced Brain Abnormalities

In addition to ethanol-induced changes in facial shape, stage of exposure-dependent alterations in brain volume and shape were examined. As shown in Fig. 3, total brain volume was significantly decreased in the GD7 ethanol exposure group. To identify disproportionate changes in regional brain volumes independent of reductions in overall brain size, the regional volume of each individual brain structure was calculated and expressed as a percentage of total brain volume. Multiple analyses of variance revealed significant differences in regional brain volumes between the various prenatal exposure groups. Post-hoc analyses indicated differences between the two ethanol exposure groups in the volumes of the olfactory bulbs, hippocampi, septal region, third ventricle, and pituitary. Further, following GD8.5 ethanol exposure, olfactory bulb, hippocampus, third ventricle, and pituitary volumes were significantly increased relative to controls, while only olfactory bulb volume was significantly reduced in the GD7 exposure group. For paired structures, volume differences in each lobe contributed comparably to overall changes (Fig. S3). Volume of the right and left cerebral cortices, lateral ventricles, and diencephalon were not significantly different in either exposure group nor was volume of mid- and hindbrain regions, which was calculated by subtracting segmented regions from total brain volume.

**Figure 6:**
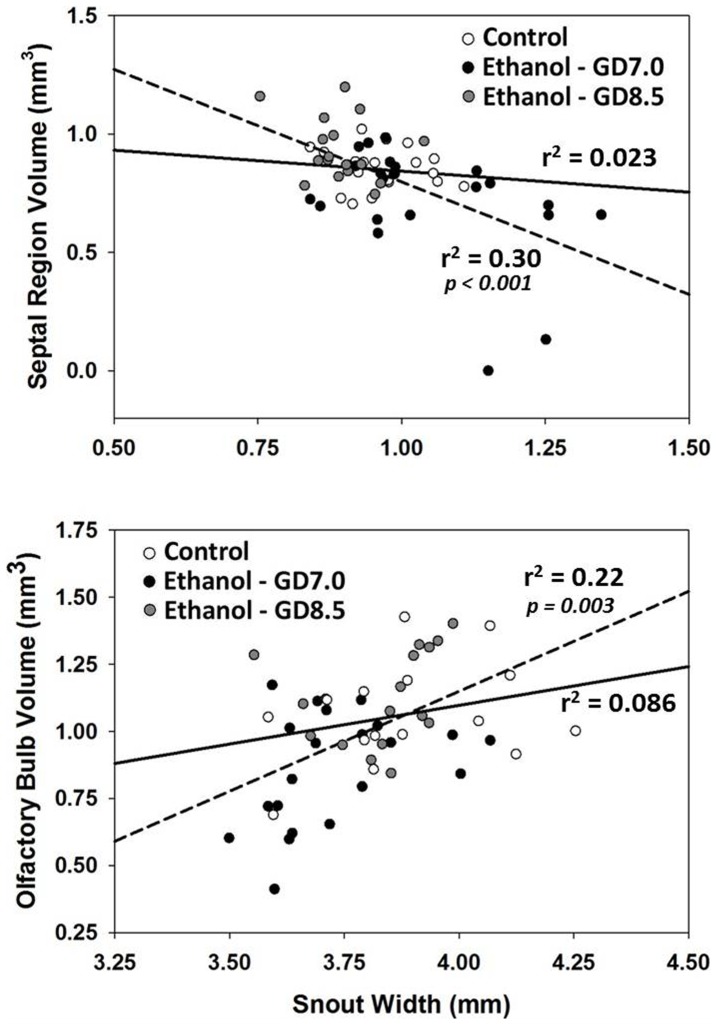
Stage-specific ethanol exposure causes correlative face and brain abnormalities. Regression lines were plotted for the control group (solid line) and for the ethanol exposure groups (dashed line). In the exposed groups, a significant negative correlation between philtrum length and septal region volume, along with a significant positive correlation between snout width and olfactory bulb volume, was found. Correlations in the control group were not significant (p = 0.55, p = 0.30, respectively).

Next, DSM was used to define shape changes contributing to overall volumetric differences in selected brain regions. Fig. 4 provides an anterior-oblique view of surface comparisons, facilitating concurrent visualization of the face along with the olfactory bulbs, septal region, and pituitary; three brain regions where robust opposing differences in the two ethanol exposure groups were evident in volumetric analyses. Apparent size differences described by surface normal comparisons are consistent with hypoplasia of the olfactory bulbs and septal region in the GD7 exposure group, and hyperplasia of the olfactory bulbs, septal region, and pituitary occurring in the GD8.5 exposure group (Fig. 4A–C).

**Figure 7:**
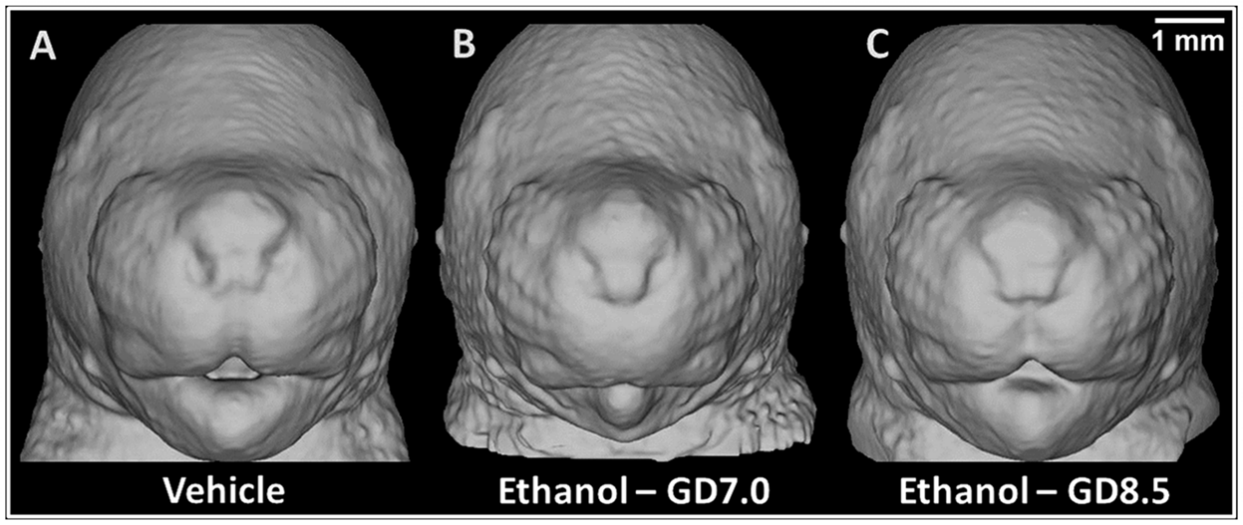
Unique facial dysmorphology induced by stage-specific ethanol exposure in the mouse corresponds to distinct clinical phenotypes. Along with a control animal (A), representative examples of fetuses severely affected by ethanol exposure on GD7 (B) and GD8.5 (C) are shown. The elongated upper lip with deficient philtrum of the GD7 exposed mouse mimics that seen in children with full-blown FAS [50]. The “fish-shaped” upper lip and bulbous nasal tip of the mouse exposed at GD8.5 resembles that of children with DiGeorge syndrome [37]. For the animals shown, the face and brain can be visualized concurrently in 3D in Movie S1.

In the GD7 exposure group, the septal region is narrow, short, and anteriorly displaced (Fig. 4D, G, J), while following exposure at GD8.5 a subtle widening and marked lengthening in the vertical direction is apparent (Fig. 4E, K). In the GD7 exposure group, slight narrowing and robust shortening in the vertical direction can be seen in the surface of the olfactory bulbs (Fig 4D, J), while in the GD8.5 exposure group, this surface is slightly widened and increased in depth (Fig. 4E, H). In both exposure groups, the pituitary gland is anteriorly displaced (Fig. 4G–I). Dynamic morphs from inferior and superior (Fig. S4), and posterior-oblique (Fig. S5) views provide complimentary illustration of important differences in regional brain shape changes associated with each ethanol exposure time point. Statistical analyses of simple linear measurements performed on all animals in each exposure group confirmed that key brain abnormalities identified in the subpopulation randomly chosen for DSM and volumetric analyses were representative of the entire study population (Fig. S6).

**Table 1:** Study population.

Treatmentgroup	LittersGenerated	Animalsscanned	Brainsegmentation	Densesurfacemodeling
Vehicle(control)	5	37	18	17
Ethanol –GD7	5	40	24	19
Ethanol –GD8.5	4	29	18	18
*Total*	*14*	*106*	*60*	*54*

To investigate the embryogenesis of identified fetal brain abnormalities, scanning electron microscopy was performed on ethanol and vehicle-exposed GD12.0 embryos (Fig. 5). In the GD8.5 ethanol-affected embryo shown, the third ventricle is markedly wider than that in the control (Fig. 5C). Viewing the anterior aspect of the brain of the embryo that was exposed to ethanol on GD7, it is apparent that the medial ganglionic eminences are too closely approximated (Fig. 5E), while in the GD8.5 ethanol-exposed embryo, they appear small and are too widely spaced (Fig. 5F). Bounded laterally by the ganglionic eminences, the developing septal region is abnormally narrow in the GD7 ethanol-treated embryo, but abnormally widened in the embryo that was exposed to ethanol on GD8.5 compared to the control embryo. Sagittal views show comparable effects, with the anterior-posterior extent of the rudimentary septal region (the forebrain territory spanning the base of the ganglionic eminence) being too short in the GD7 ethanol-exposed embryo (Fig. 5H), while in the GD8.5 ethanol-exposed embryo, it is too long (Fig. 5I).

### Correlative Face and Brain Phenotypes

The DSM-based shape analysis described above demonstrates that developmental stage-specific ethanol exposure results in distinct facial phenotypes that correspond to unique brain abnormalities. However, comparisons of mean values do not capture how these changes correlate across the spectrum of severity present in each group. Therefore, the relationship between selected face and brain features was examined across the range of phenotypic severity. Among animals exposed to ethanol prenatally, there was a significant negative correlation between median upper lip length and septal region volume and a positive correlation between snout width and olfactory bulb volume (Fig. 6). In contrast, no significant correlations were detected between these variables among controls.

## Discussion

The integration of advanced technologies for small animal imaging and shape analysis implemented here represents an innovative tool for defining concurrent abnormalities of the face and brain in 3D. The MRM imaging parameters utilized provided a high-throughput capability (35 min scan/animal), allowing examination of a large number of animals encompassing the phenotypic range resulting from each of two acute ethanol exposure times. Acquired 3D image sets allowed segmentation of regional brain structures as well as extraction of remarkably accurate and detailed facial surfaces from fetal mice measuring just 15 mm from the crown to rump. In addition to linear and volumetric measurements, this enabled application of DSM and assessment of 3D changes in facial surfaces and brain regions. While this technique has proven useful in modeling and delineating face shape differences across populations with various genetic syndromes, this study represents its first application in defining concurrent face-brain shape changes. These results will be readily comparable to the findings of ongoing clinical studies using DSM to define facial shape in populations with FAS, as well as those not meeting diagnostic criteria but having documented heavy prenatal ethanol exposure [33].

The results of this study have important conceptual and practical implications for diagnostic and interventional strategies in FASD. Conceptually, they demonstrate that early prenatal ethanol exposure can cause more than one pattern of facial dysmorphology. Practically, these results should encourage new avenues for clinical FASD research by highlighting abnormalities of specific facial features and brain regions that have previously received little attention. The pattern of craniofacial abnormalities resulting from insult during early gastrulation, including microcephaly, shortened palpebral fissures and an elongated upper lip with a smooth/deficient philtrum, is consistent with that currently recognized for FAS. Median forebrain deficiencies have been well described in populations with FAS [23], [25], [34], [35], and recent MRI studies have demonstrated that palpebral fissure length is significantly correlated with abnormal cortical thickness and corpus callosum reduction [23], [24]. The correlation data presented here extend these findings by suggesting that the type and severity of brain abnormalities can be predicted, in part, by dysmorphology of the upper lip and hypoplasia of the midface.

Among the GD7 ethanol-exposed subjects, increased upper lip length was associated with hypoplasia of the olfactory bulbs and septal region. Remarkably, ethanol insult just 1.5 days later yielded a unique, and partially opposing pattern of dysmorphic features, including an abnormally shortened upper lip associated with increased septal region volume. As illustrated in Figs. 7 and Movie S1, and as previously suggested [36], the GD8.5 ethanol-induced facial dysmorphia appear more similar to those in individuals with DiGeorge syndrome (OMIM# 188400) [37]. This malformation sequence commonly results from a 22q11.2 deletion but has also been reported in humans as a consequence of prenatal ethanol exposure [38]. Individuals with DiGeorge syndrome and FASD also share a substantially increased incidence of depression and psychotic disorders [39], [40]. Particularly notable are findings relative to schizophrenia, which is common in DiGeorge syndrome and also occurs as part of FASD [39], [41]. Clinical imaging studies have shown an increased incidence of cavum septum pellucidum in adults with schizophrenia, as well as in those with DiGeorge syndrome [42], [43], [44]. These investigations have not focused on the septal region, per se. However, its developmental and anatomical relationship to the septum pellucidum [45], along with the results of the current study, which provide a testable hypothesis to explore the underlying source of neurobehavioral phenotypic variation associated with FASD, indicate that such analyses are warranted.

Cumulatively, the results of this study provide a proof of principle that early prenatal ethanol exposure can cause more than one temporally-dependent pattern of defects involving the face and brain and illustrate the predictive nature of the facial features for associated brain abnormalities. Numerous mechanisms for ethanol teratogenesis have been proposed. While outside the scope of the work presented here, the finding that ethanol elicits stage-specific teratogenic effects should provide new opportunities to examine these mechanisms within specific embryological contexts. Of clinical importance, these results suggest that an expansion of current diagnostic criteria to better represent the range of facial phenotypes induced by prenatal ethanol exposure could significantly advance early diagnosis and intervention strategies that are critical for the management of FASD.

## Materials and Methods

### Animals and Ethanol Administration

All animal treatment protocols were approved by the University of North Carolina at Chapel Hill Institutional Animal Care and Use Committee. C57Bl/6J mice were purchased from The Jackson Laboratory. Timed-pregnancies were established by housing two female mice with one male mouse for a period of two hours. GD0 was defined as the beginning of the breeding period in which a copulation plug was found. Pregnant dams were administered two 25% ethanol (v/v in lactated Ringer’s solution) dosages of 2.9 g/kg by ip injection four hrs apart beginning at GD7 or GD8.5. Blood ethanol concentrations resulting from this exposure paradigm have already been described [19], [46]. Control animals were injected with an equivalent volume of lactated Ringer’s solution. GD17 fetuses were prepared for MRM as previously described (16).

### Magnetic Resonance Microscopy

MRM was performed on a 9.4 T vertical bore magnet interfaced to a GE console running Epic 12.4X (GE Medical Systems). The system is equipped with Resonance Research gradients (Resonance Research, Inc.), which achieve peak gradients of 2000 mT/m. Two fetal heads were placed in an acrylic sample holder and immersed in fomblin. 3D volume images were acquired in a 10 mm diameter×25 mm long solenoid radiofrequency coil using a radiofrequency refocused spin echo sequence (TR = 50 ms, TE = 5.2 ms, field of view = 20×10×10 mm, matrix size = 512×256×256), resulting in isotropic spatial resolution of 39 µm. A novel acquisition strategy that amplifies the high-frequency information by selectively altering the receiver gain during the phase-encoding steps was applied to extend the dynamic range of the system, capture the higher-frequency components, and limit saturation in the central k-space [47]. Total scan time for each pair of specimens was approximately 1.1 hrs.

### Brain Segmentation

Forebrain and pituitary regions were manually segmented using ITK-Snap (Version 2.1.4) as previously described [18]. Automated skull-stripping [48] was used to generate whole brain volumes. Since no external brain atlas matching the population was available, a reference image was randomly chosen between cases to generate a probabilistic tissue segmentation atlas. This case was manually skull-stripped and intensity thresholded to create three categories coarsely representing the white matter, gray matter and an intermediate category. ABC, a tool that implements the Expectation-Maximization Segmentation algorithm, was used to classify the different tissues of the brain, which were subsequently combined to generate whole-brain masks. The largest connected component of the obtained segmentation was selected to remove voxels outside the brain wrongly classified and a binary closing operation was performed to smooth the boundaries and get rid of small regions erroneously classified as non-brain.

### Linear and Volumetric Measurements

Linear facial measurements were obtained from 3D head reconstructions using netfabb Studio Basic (Version 4.7). Volumetric brain measurements were obtained with ITK-Snap.

### Dense Surface Modeling

DSM was performed as previously described [26], [49] using anthropometric landmarks adapted for the mouse. For each individual image, the following anthropometric facial landmarks were manually marked; left-right paired alare, cheilion, endocanthus, exocanthus, lower/upper ear attachment, philtral pillar; left-right paired primordia of vibrissae (supraorbit, infraorbit, gonial, upper and lower extremes of the muzzle); upper lip center, lower lip center, nasion, pronasale, subnasale, gnathion. For brain regions, five midline and nine left-right paired points were used as indicated in Fig. S7. These landmarks were used to guide the formation of a dense correspondence between a common set of points across all face and brain surfaces in each treatment group as previously described [26], [49].

### Linear Brain Measurements

Linear brain measurements were obtained from MRM sections using ImageJ (Version 1.43; http://rsb.info.nih.gov/ij/).

### Histology

Following MRM imaging, fetuses fixed in Bouin's solution were transferred to 70% ethanol for at least two weeks. After paraffin embedding, 10 µm sections were produced and stained with hematoxylin and eosin by standard protocols.

### Statistics

Multivariate analyses of variance (MANOVAs) were used to determine significant linear and volumetric group differences. Significant between-subject effects were followed by Student Newman Keuls posthoc tests when appropriate. To explore the relationship between regional brain volumes and facial linear measurements, Pearson’s correlation coefficients were utilized. An alpha value of 0.05 was maintained for all analyses.

### Study Population

For each treatment group, the number of litters and total fetuses produced is listed in Table 1. From each litter, all fetuses were imaged by MRM to facilitate examination of a spectrum of severity. For comparison with both ethanol treatment groups, the control population was comprised of five vehicle-treated litters, including three exposed at GD7 and two exposed at GD8.5. Due to the extensive time required, approximately half of all imaged animals from each group were randomly selected for manual brain segmentation. To examine correlative face and brain phenotypes, DSM was performed on this sub-population. In a few cases, signal artifact surrounding the facial surface precluded inclusion in DSM analysis. Linear brain measurements and ocular defect analysis were performed on all scanned animals.

## Supporting Information

Figure S1 **Morphing illustrates unique facial phenotypes in each ethanol exposure group.** Rapidly interpolated images provide dynamic morphs between mean control (WT) and mean ethanol-exposed facial surfaces in portrait and profile view.
(GIF)

Figure S2 **Stage-specific ethanol exposure causes varying degrees of ocular defects.** Prior to fixation of vehicle and ethanol exposed fetuses for MRM, both eyes were imaged by bright-field microscopy. Ocular defects were rated on a scale from 1–5 as follows: (1) normal; (2) slight microphthalmia or slight pupil shape abnormality; (3) slight microphthalmia and slight pupil shape abnormality, (4) moderate microphthalmia; and (5) severe microphthalmia and as previously described [30]. Representative images of each defect category are shown below. Analysis was performed in the entire study population.
(TIF)

Figure S3 **Volumetric analysis of individual brain regions in control and ethanol exposure groups.** Individual brain region volumes were derived from manual segmentation. To determine disproportionate differences, the volume of each region was calculated as a percentage of total brain volume in each animal. Paired structures are shown individually. Letters above each bar indicate group classes; the same letter above a subset of bars denotes lack of statistical difference, whereas different letters represent statistically different classes (p<0.05).
(TIF)

Figure S4 **Morphing illustrates unique brain and facial phenotypes in each ethanol exposure group.** Rapidly interpolated images provide dynamic morphs between the mean control (WT) and mean ethanol-exposed brain and facial surfaces from a superior (downward at the snout) and inferior (upward at the mandible) view.
(GIF)

Figure S5 **Morphing illustrates opposing changes in brain region shape between ethanol exposure groups.** Rapidly interpolated images provide dynamic morphs between the mean GD7 exposure group and the mean GD8.5 exposure group from a posterior-oblique view.
(GIF)

Figure S6 **Linear brain measurements of entire populations reflect volumetric and DSM results.** Linear measurements were produced from transverse MRM sections at the level of the anterior commissure in all scanned animals. Biparietal diameter, spanning the widest distance across the cerebrum, third ventricular width (TVW), and septal region width (SRW) were measured. For each group, individual litters are represented by separate columns of data points, which are superimposed on box and whisker plots. Box parameters represent the 25th and 75th percentile of the population, while error bars represent the 10th and 90th percentile. Inside the boxes, a solid line represents the population median, while the mean is represented by a dashed line. Letters above each bar indicate group classes; the same letter above a subset of bars denotes lack of statistical difference, whereas different letters represent statistically different classes (p<0.05). The septal region was absent (%) at the level of the anterior commissure in three animals exposed to ethanol at GD7. These values are not plotted, nor included in population statistics. H&E stained sections from an animal in each treatment group illustrate the septal region area corresponding to the boxed region in the MRM section.
(TIF)

Figure S7 **Landmarks used for DSM analysis of selected brain regions are shown in superior and inferior views.**
(TIF)

Movie S1 **3D visualization of face-brain dysmorphology resulting from stage-specific ethanol exposure.** For the animals shown in Figure 7, the brain and face are visualized concurrently in 3D by reducing head surface opacity using Slicer3, an open source image visualization platform (www.slicer.org).
(WMV)
