# Supplementary figures and images for: Ethanol-Induced Face-Brain Dysmorphology Patterns Are Correlative and Exposure-Stage Dependent
Source: PLoS One. 2012 Aug 22;7(8):e43067. doi: 10.1371/journal.pone.0043067 (PMC3425589; doi:10.1371/journal.pone.0043067)

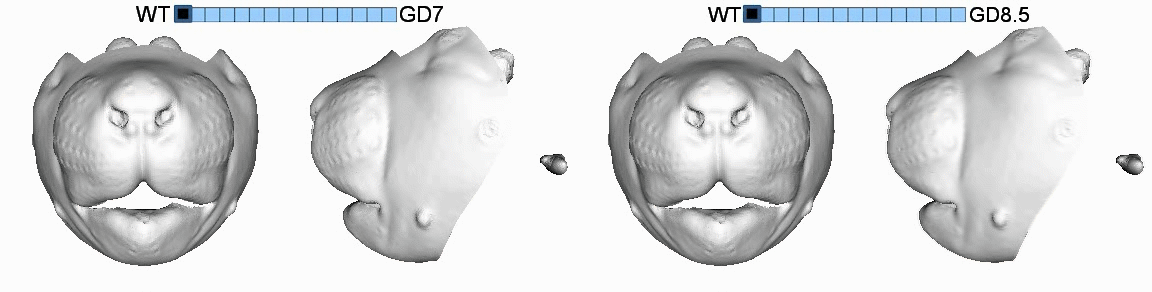

Supplement: Figure S1 — Morphing illustrates unique facial phenotypes in each ethanol exposure group. Rapidly interpolated images provide dynamic morphs between mean control (WT) and mean ethanol-exposed facial surfaces in portrait and profile view. (GIF) [file pone.0043067.s001.gif]

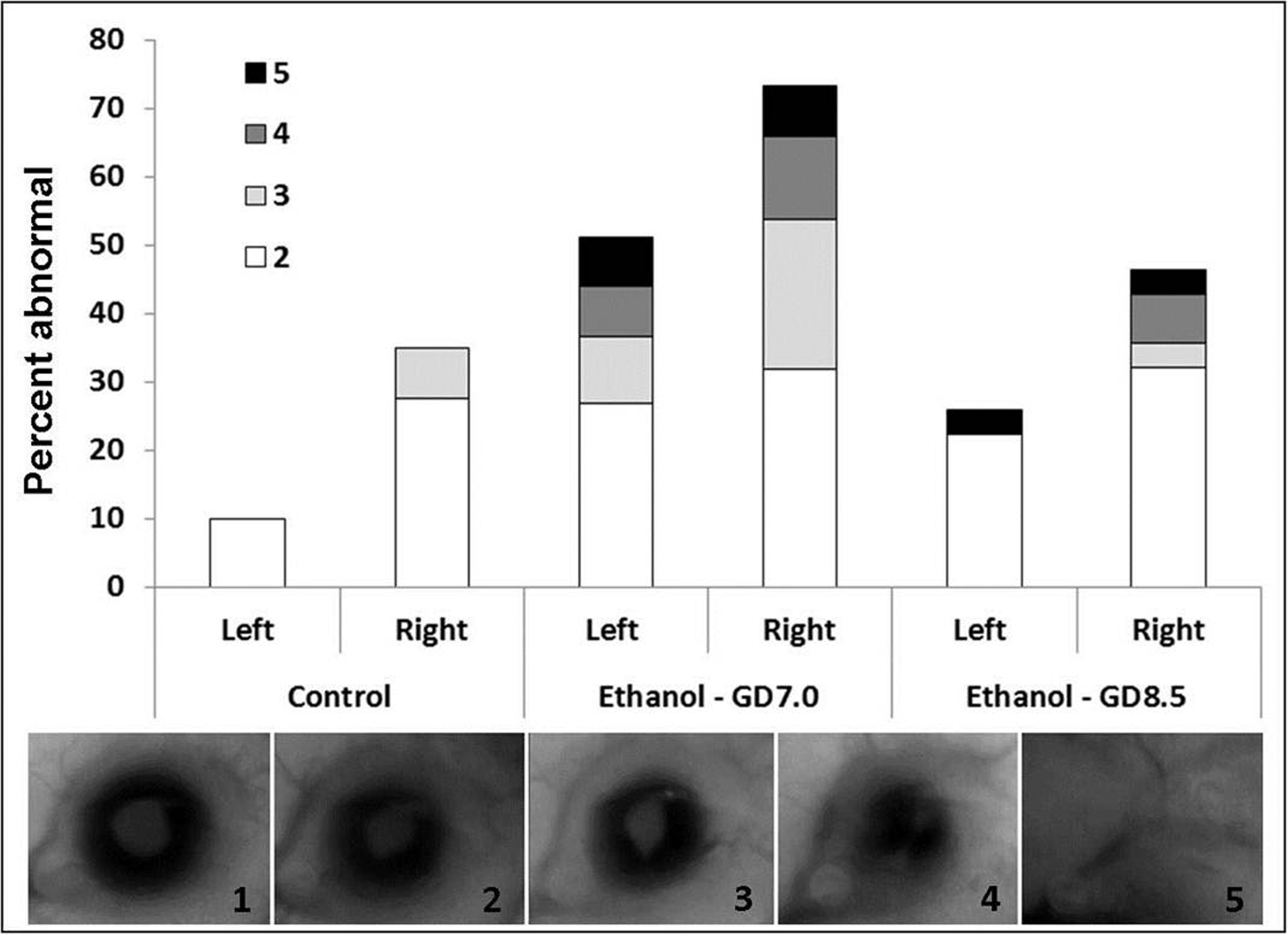

Supplement: Figure S2 — Stage-specific ethanol exposure causes varying degrees of ocular defects. Prior to fixation of vehicle and ethanol exposed fetuses for MRM, both eyes were imaged by bright-field microscopy. Ocular defects were rated on a scale from 1–5 as follows: (1) normal; (2) slight microphthalmia or slight pupil shape abnormality; (3) slight microphthalmia and slight pupil shape abnormality, (4) moderate microphthalmia; and (5) severe microphthalmia and as previously described [30]. Representative images of each defect category are shown below. Analysis was performed in the entire study population. (TIF) [file pone.0043067.s002.tif]

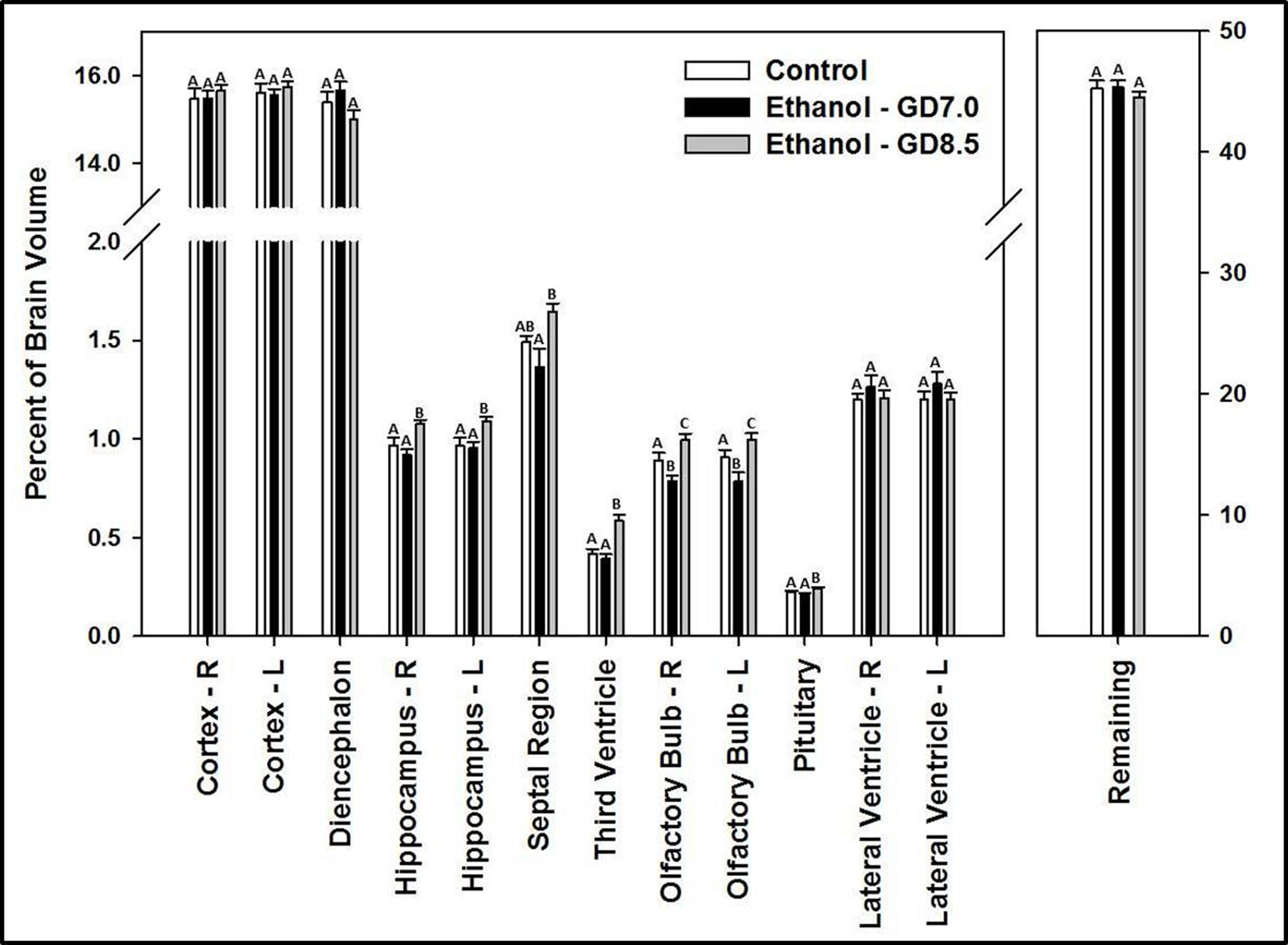

Supplement: Figure S3 — Volumetric analysis of individual brain regions in control and ethanol exposure groups. Individual brain region volumes were derived from manual segmentation. To determine disproportionate differences, the volume of each region was calculated as a percentage of total brain volume in each animal. Paired structures are shown individually. Letters above each bar indicate group classes; the same letter above a subset of bars denotes lack of statistical difference, whereas different letters represent statistically different classes (p<0.05). (TIF) [file pone.0043067.s003.tif]

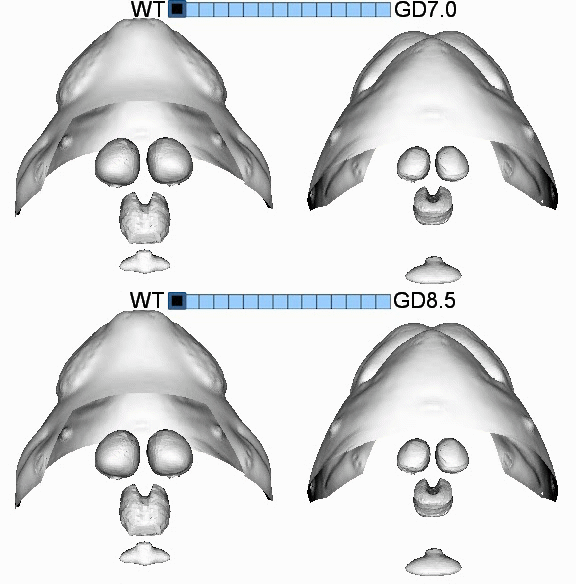

Supplement: Figure S4 — Morphing illustrates unique brain and facial phenotypes in each ethanol exposure group. Rapidly interpolated images provide dynamic morphs between the mean control (WT) and mean ethanol-exposed brain and facial surfaces from a superior (downward at the snout) and inferior (upward at the mandible) view. (GIF) [file pone.0043067.s004.gif]

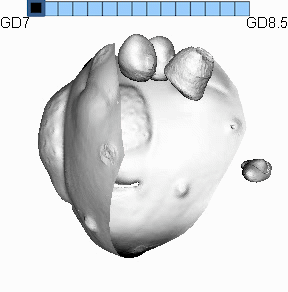

Supplement: Figure S5 — Morphing illustrates opposing changes in brain region shape between ethanol exposure groups. Rapidly interpolated images provide dynamic morphs between the mean GD7 exposure group and the mean GD8.5 exposure group from a posterior-oblique view. (GIF) [file pone.0043067.s005.gif]

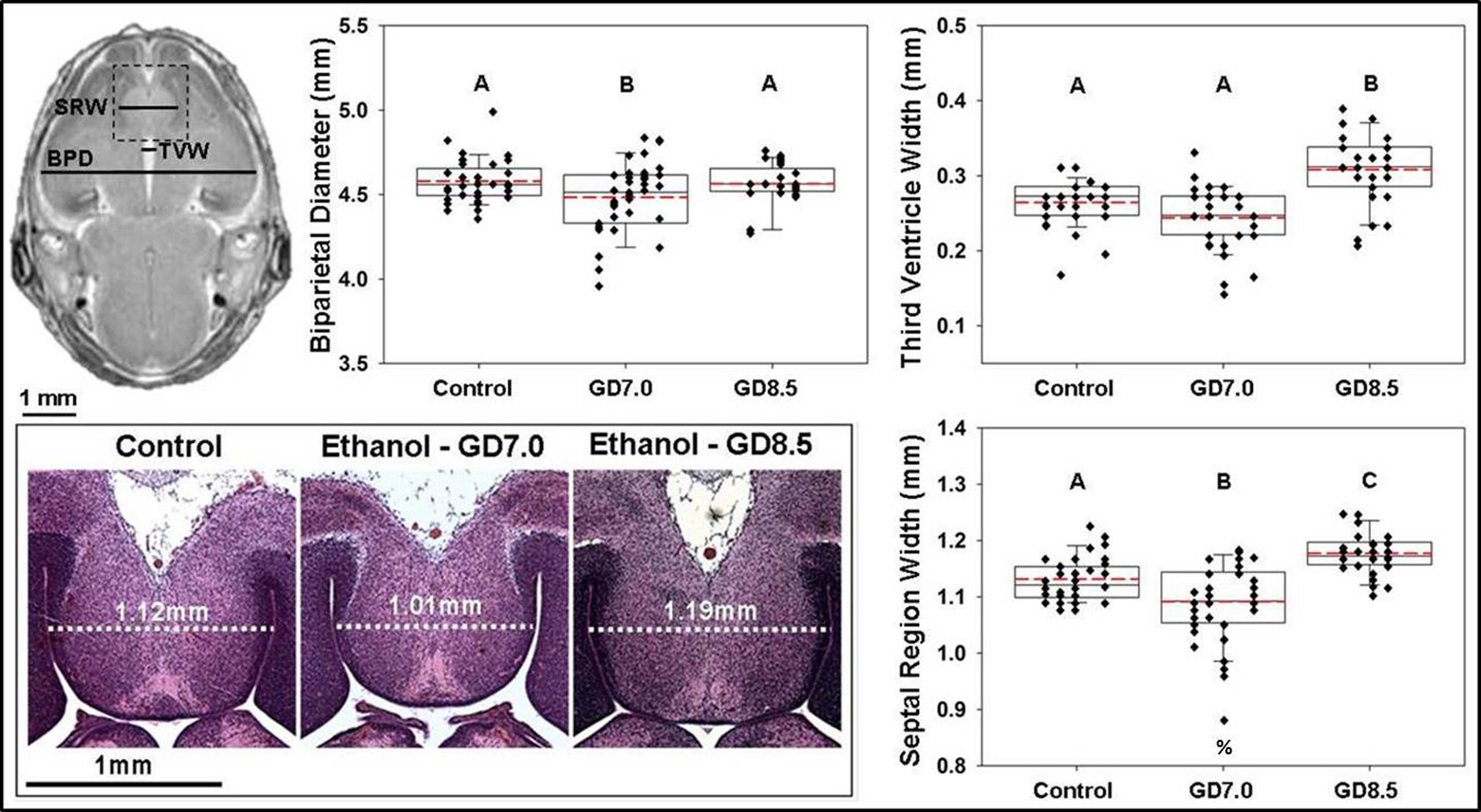

Supplement: Figure S6 — Linear brain measurements of entire populations reflect volumetric and DSM results. Linear measurements were produced from transverse MRM sections at the level of the anterior commissure in all scanned animals. Biparietal diameter, spanning the widest distance across the cerebrum, third ventricular width (TVW), and septal region width (SRW) were measured. For each group, individual litters are represented by separate columns of data points, which are superimposed on box and whisker plots. Box parameters represent the 25th and 75th percentile of the population, while error bars represent the 10th and 90th percentile. Inside the boxes, a solid line represents the population median, while the mean is represented by a dashed line. Letters above each bar indicate group classes; the same letter above a subset of bars denotes lack of statistical difference, whereas different letters represent statistically different classes (p<0.05). The septal region was absent (%) at the level of the anterior commissure in three animals exposed to ethanol at GD7. These values are not plotted, nor included in population statistics. H&E stained sections from an animal in each treatment group illustrate the septal region area corresponding to the boxed region in the MRM section. (TIF) [file pone.0043067.s006.tif]

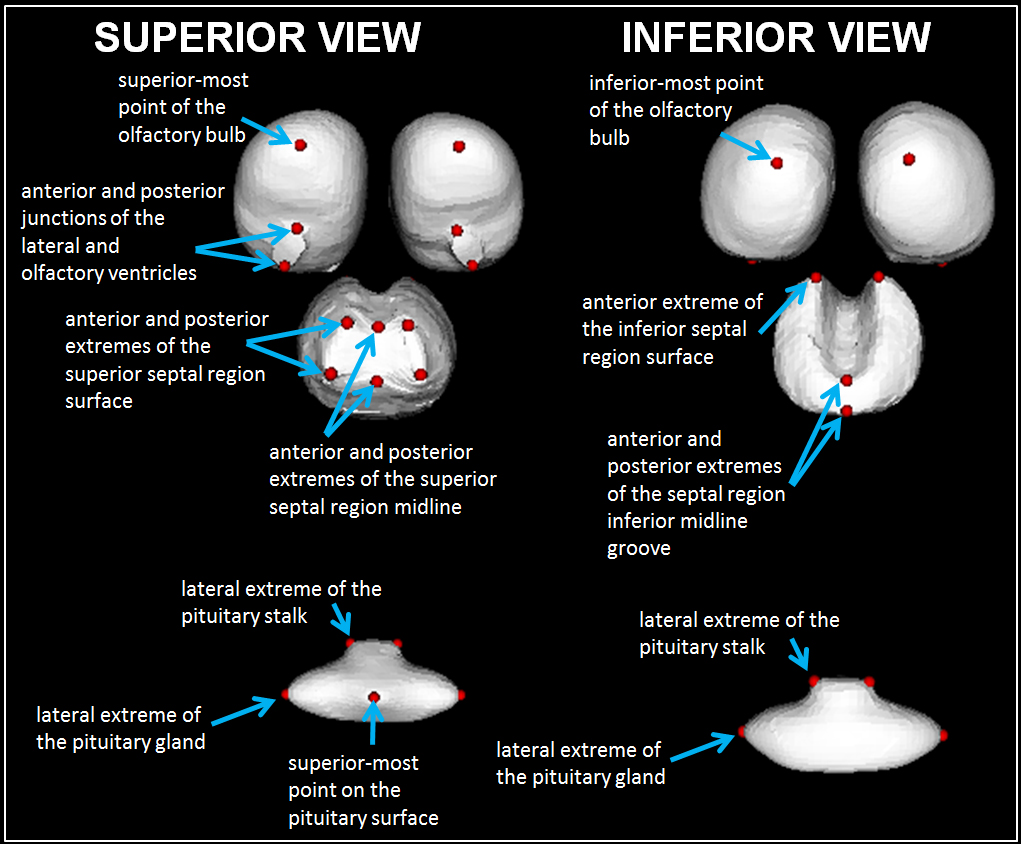

Supplement: Figure S7 — Landmarks used for DSM analysis of selected brain regions are shown in superior and inferior views. (TIF) [file pone.0043067.s007.tif]
